# Pro-Inflammatory Cytokines as Early Predictors of Chronic Rheumatologic Disease Following Chikungunya Virus Infection

**DOI:** 10.3390/jcm14196720

**Published:** 2025-09-23

**Authors:** Alessandro Conforti, Gabriella Lavalle, Farbizio Varini, Linda Lucchetti, Giulia Cataldi, Augusto Faticoni, Marco Ruggiero, Martina Gentile, Giancarlo Gimignani, Matteo Bassetti

**Affiliations:** 1 Rheumatology Health Care, ASL Roma 4, 00053 Civitavecchia, Italy; 2 UOC Patologia Clinica, P.O. Bracciano, ASL Roma 4, 00062 Bracciano, Italy; gabriella.lavalle@aslroma4.it; 3 Department of Marine and Biology and Ecology, University of Tuscia, 01100 Viterbo, Italy; varini.fabrizio@unitus.it; 4 Hospital Pharmacy, San Paolo Hospital, ASL Roma 4, 00053 Civitavecchia, Italy; linda.lucchetti@aslroma4.it; 5 Health Care, ASL Roma 4, 00053 Civitavecchia, Italy; 6 National Institute for Infectious Diseases “Lazzaro Spallanzani”—IRCCS, 00149 Rome, Italy; faticoniaugusto@gmail.com; 7 IRCCS Istituto Ortopedico Rizzoli, 44011 Argenta, Italy; 8 Health Care, ASL Viterbo, 01100 Viterbo, Italy; martinagnt@gmail.com; 9 UOC Internal Medicine, San Paolo Hospital, ASL Roma 4, 00053 Civitavecchia, Italy; giancarlo.gimignani@aslroma4.it; 10 Infectious Diseases Clinic, Policlinico San Martino Hospital—IRCCS, 16132 Genoa, Italy; matteo.bassetti@unige.it

**Keywords:** arthralgia, arthritis, chikungunya fever, cytokines, interferons, musculoskeletal diseases

## Abstract

**Background/Objectives:** Infection with the Chikungunya virus (CHIKV) results in acute febrile diseases with severe polyarthritis, which can be followed by chronic rheumatic disease and long-term disability. To evaluate cytokine profiles in acute and chronic CHIKV infection and examine the relationship with clinical outcomes, including persistent musculoskeletal symptoms. **Methods:** Literature search was carried out systematically using electronic databases (PubMed, Embase, Scopus, and Google Scholar). Studies that met eligibility criteria were observational or intervention cohorts that measured levels of cytokines/chemokines in laboratory-confirmed CHIKV patients in acute (<3 months) or chronic (>3 months) phases. Data was extracted on the study design, population, biomarkers, and clinical outcomes. As the assays were not homogeneous in terms of timing, outcome, and definitions, the findings were synthesized in narrative form using tabular representation. **Results:** A total of 21 studies, including 4216 participants, were incorporated. In acute CHIKV, interleukin-6 (IL-6) (reported to be elevated in 81% of studies), IL-8 (77%), TNF-α (65%), and IFN-γ (60%) were most consistently increased, alongside interferon-inducible chemokines CXCL10/IP-10 and MCP-1. Chronic-phase cohorts showed persistent elevation of IL-6 (69%), IL-8 (63%), TNF-α (58%), and IL-17 (41%), especially among patients with prolonged arthralgia or arthritis. Chronic musculoskeletal symptoms were reported in 25–65% of cases, with some persisting beyond five years. A high degree of heterogeneity was observed in assay procedures and time of sampling. **Conclusions:** CHIKV infection is characterized by acute inflammatory surges and, in some cases, persistent cytokine dysregulation linked to chronic arthritis. These results showed the importance of prognostic biomarkers and targeted therapy.

## 1. Introduction

Chikungunya virus (CHIKV) is an arthritogenic alphavirus, which has spread globally since 2004, with explosive outbreaks associated with fever and severe polyarthralgia, and leaving a large proportion of survivors with extensive long-term musculoskeletal morbidity [1]. In reviews and cohort studies, it has been demonstrated that chronic or recurrent arthralgia is frequent and disabling, and that it has implications for the quality of life and economic burden. Mechanistic and epidemiologic syntheses underscore the fact that CHIKV musculoskeletal disease is inflammatory in nature and may continue to persist long after the viremic phase, highlighting the importance of early predictors of chronic outcomes [2].

This burden is measured in longitudinal studies. In a 36-month, prospective longitudinal study (*n* = 180), 60% of the patients developed recurrent or persistent arthralgia that was most frequently symmetrical and incapacitating; the predictor of longer-term pain was age > 35 years and symptoms at 4 months [2]. In previous post-epidemiologic follow-up studies, 57% reported rheumatic symptoms 15 months later, and non-recovery was linked to older age and severe initial joint pain [3]. These are estimates learned from various outbreaks and environments demonstrating a risk that can be repeated following CHIKV infection, which must lead to chronic rheumatologic disease.

The study of pathogenesis focuses on the role of viral persistence and the inflammatory response of the host as contributors to chronicity. CHIKV RNA and proteins have been reported in perivascular synovial macrophages as long as 18 months after infection in humans with chronic disease and with continuous foci of inflammatory infiltrates [4]. Long-term retention of viruses in tissue macrophages in the nonhuman primate model supports long-term persistence of inflammation and pain in joints after the acute illness and gives it biological plausibility [5]. Collectively, this data supports a model where the main source of infection seeds the tissue, where the ongoing antigenic stimulation maintains pathology.

Profiling of cytokines throughout the disease process supports the idea that certain soluble mediators could mediate the passage of acute events to chronic consequences. In acute infection, increased viremia is consistent with increased interferon-α and interleukin-6 (IL-6), as well as with lymphopenia and neutrophilia, which are hallmarks of increased systemic inflammation. Notably, a classic clinical study connected persistent arthralgia with increased acute-phase IL-6 and granulocyte–macrophage colony-stimulating factor (GM-CSF), as well as complete recovery with increased eotaxin and hepatocyte growth factor [6]. Follow-up studies and cross-sectional data have implicated IL-17, IL-27, and other pro-inflammatory cytokines in individuals with persistent symptoms and have supported an immunologic signature associated with chronic sequelae [7].

These findings are synthesized in meta-analyses and narrative reviews. A review of the existing literature on the immune signatures of CHIKV victims shows the consistent links between high levels of pro-inflammatory cytokines, especially IL-6 and GM-CSF, and persistent arthralgia and the heterogeneity in the timing, assays, and definition of cases among studies [8]. Broader reviews map the shift in acute interferon-induced responses to chronic and inflammation localized to the joints, address macrophage reservoirs, and highlight the gaps in validated prognostic biomarkers that can be measured at the acute stage to predict the long-term results [9].

The clinical implications of the identification of early biomarkers of chronic rheumatologic disease following CHIKV have direct implications. Chronic chikungunya arthritis has similar inflammatory pathways to other arthritides (e.g., with a central role in IL-6 and Th17-related cytokines), but differs in autoantibody profiles and, in many cases, is not accompanied by classical signs of systemic autoimmunity [10,11,12]. The identification of patients at increased risk at the outset would help to refine the counseling, intensity of follow-up, and prompt implementation of anti-inflammatory measures in resource-restricted epidemic environments in which the cost of persistent pain and disability is high.

This review study aims to determine whether pro-inflammatory cytokines at the acute stage of CHIKV infection are predictive of the onset of chronic rheumatologic disease. Based on findings that acute IL-6 and GM-CSF elevations are correlated with either persistent or transient arthralgia and that cytokines like IL-17/IL-27 are predictive of chronic symptomatology, we will assess mediators at the viremic/early convalescent periods and correlate them with standardized long-term musculoskeletal outcomes. The development of strong and early immunologic predictors would represent a key gap in chikungunya treatment and would potentially be used to inform risk-stratified pathways during future epidemics.

## 2. Materials and Methods

### 2.1. Search Strategy

We carried out an extensive electronic database (PubMed, Embase, and Scopus) and gray literature (Google Scholar) search. No language restriction was applied to the search, and all years up to 2nd September 2025 were covered.

Combined search terms were as follows: controlled vocabulary and keywords (MeSH terms) involving Chikungunya virus, cytokines, pro-inflammatory mediators, arthralgia, arthritis, and chronic rheumatologic disease. The query was refined using the Boolean operators (AND/OR/NOT).

### 2.2. Eligibility Criteria

Our eligibility criteria were based on the PICO framework. Human participants who were confirmed to be infected by CHIKV through PCR or serology were included in the population of interest. Measurement of pro-inflammatory cytokines, including IL-6, TNF-α, IL-1β, IFN-γ, and IL-17 during the acute infection phase, was the eligible intervention. They compared patients with high and non-high levels of cytokines, patients with developed chronic rheumatologic disease, and patients with complete recovery. Studies were also eligible that reported musculoskeletal disease chronic outcome defined as persistent arthralgia, arthritis, or musculoskeletal disability with a follow-up time of three months or greater. We limited our search to excluding animal research, in vitro research, case reports, reviews, editorials, commentaries, and research that did not involve measurements of cytokines or outcomes.

### 2.3. Study Selection

**Figure 1:**
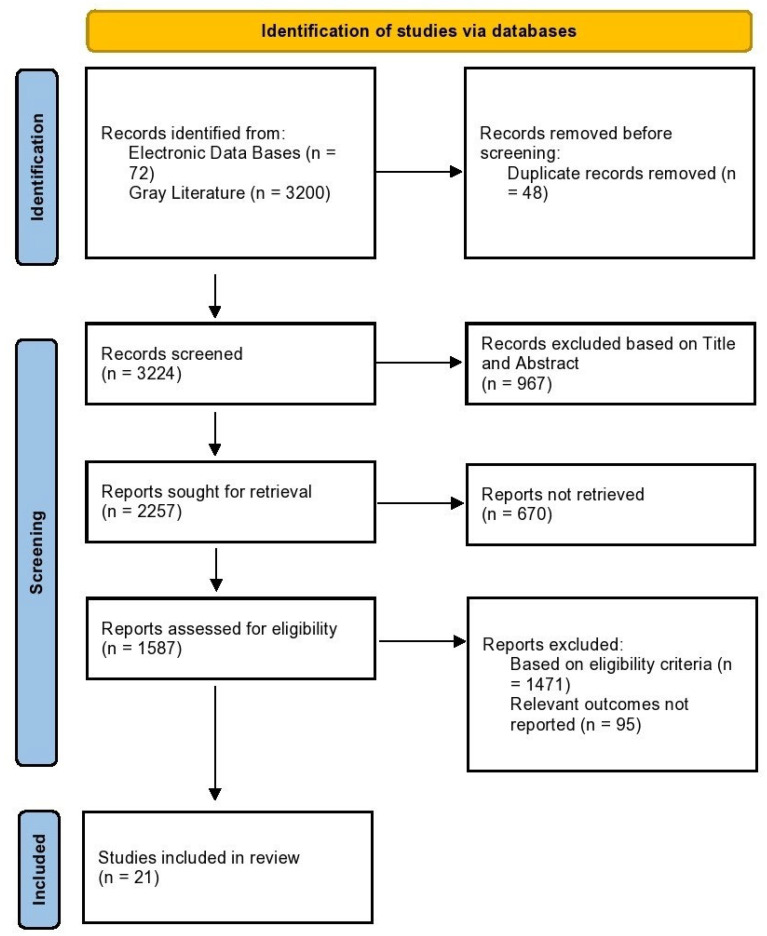
PRISMA flow diagram.

All retrieved records were imported into EndNote software 21 software in study citation were imported during de screening, followed by the removal of duplicates. Titles and abstracts were also considered in the initial step in order to exclude records that were irrelevant. The next step was the retrieval and assessment of full-text articles of potentially eligible studies using the inclusion and exclusion criteria. The reviewers disagreed and settled the dispute by discussion, and finally, 21 articles were included. This study was conducted according to Preferred Reporting Items for Systematic Reviews and Meta-Analyses (PRISMA), and the outcome of the selection process was recorded in a PRISMA flow diagram (Figure 1).

This systematic review has been registered in the PROSPERO database (Registration code: CRD420251136984).

### 2.4. Data Extraction

Two reviewers collected the data separately on a standardized extraction form (in Microsoft Excel) designed to be used in this review. The variables of interest were collected. Differences in the extraction of the data were resolved through discussion or through appeal to a third reviewer.

### 2.5. Quality Assessment and Risk of Bias

The methodological quality of all included studies was assessed using the ROBINS-I tool for non-randomized studies and visualized with the Robvis web application [13].

**Figure 2:**
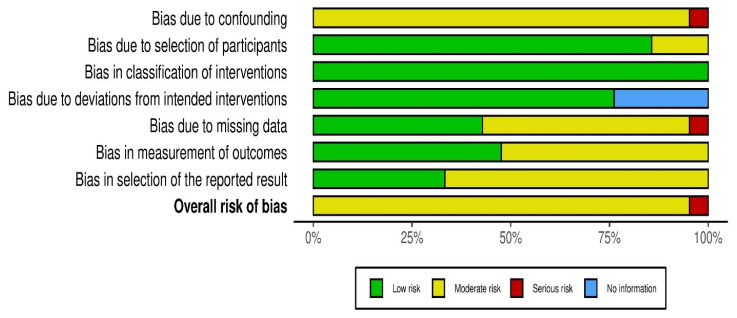
Risk of bias summary plot.

Figure 2 gives an overview of the risk of bias of all studies. An increased percentage of studies were concluded as being at serious risk of bias because of the confounding that is inherent in non-randomized study designs. Multiple studies presented moderate-to-severe issues in participant selection and interventions classification that can impact internal validity. However, studies with fewer risks were those with less reporting bias and outcome measurement risk, which implies relatively high methodological rigor.

**Figure 3:**
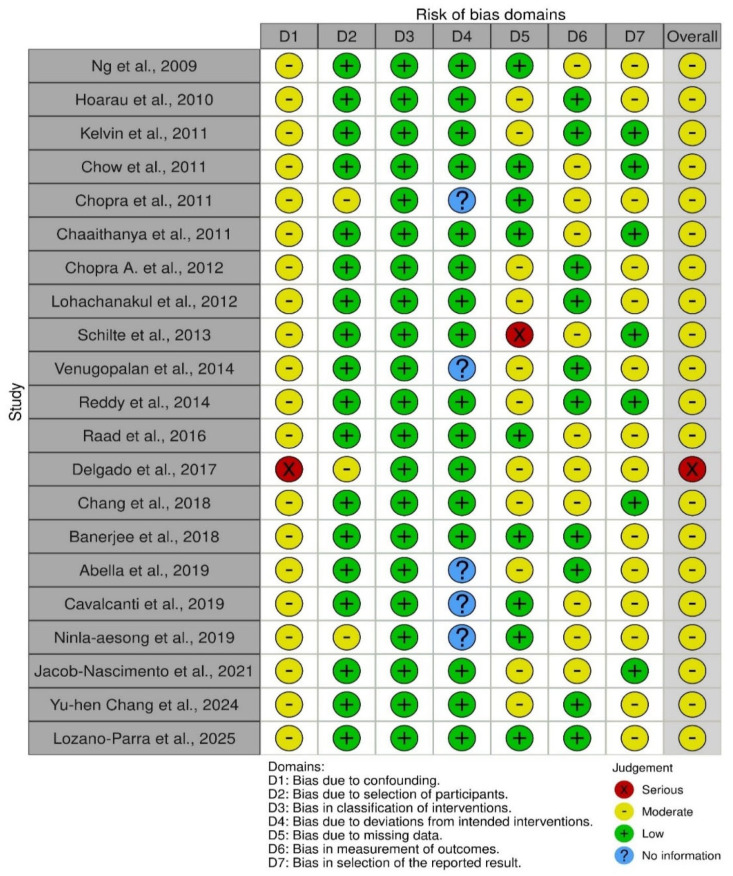
Risk of bias traffic light plot [2,4,6,7,14,15,16,17,18,19,20,21,22,23,24,25,26,27,28].

Figure 3 (the traffic light plot) additionally points out the variation in risk of bias in individual studies and domains. Some studies reported lower levels of concern in areas such as bias due to missing data and bias in measurement of outcomes, but other studies had serious or critical risk in areas such as bias due to confounding and bias in selection of participants.

## 3. Results

We included 21 studies in this review [2,4,6,7,14,15,16,17,18,19,20,21,22,23,24,25,26,27,28,29,30,31], and the studies examined the clinical and demographic characteristics of CHIKV-infected patients. Sample sizes were also considered to be quite diverse, ranging from 10 [14,21] to 509 [31], with some studies incorporating healthy or disease controls. The study designs were diverse (cohort *n* = 7 [2,4,15,19,26,27,30]; case–control *n* = 5 [6,7,22,23,28]; prospective *n* = 4 [18,20,21,31]; cross-sectional *n* = 4 [16,17,24,25]; and retrospective *n* = 1 [14]), and the follow-up periods were between 2 weeks [14] and 7.7 years [28]. The median or average age of the participants was most often reported in the period between the third and the fifth decade of life, although many studies involved old age populations (>70 years) [2]. The sex distribution in the studies was predominantly female, with some studies including up to 89% female [20,22], and only a small number of studies had a predominantly male group [6,14,19] (Table 1).

Frequently described acute manifestations in the included studies were fever, arthralgia, and myalgia, with fever being the most common in almost all patients (approximately 90%). Rash occurred in about 30–70% of cases; other frequent manifestations were headache (40–70%), gastrointestinal (20–30%), fatigue (40–80%), and conjunctivitis (5–30%). A large percentage of patients (10% to more than 50% according to study definitions such as high-grade fever, tachycardia, thrombocytopenia, or high viral load [4]) reported severe disease. Rare complications were those that involved the nervous system (e.g., meningoencephalitis) [20] (Table 2).

Patients with CHIKV infection consistently showed elevated levels of multiple pro-inflammatory cytokines compared to controls, particularly IL-6, IL-8, TNF-α, IFN-γ, MCP-1/CCL2, and CXCL9/10, with concentrations often exceeding control values by 5- to 50-fold during the acute phase. IL-6 and IL-8 were the most frequently reported markers [26], with acute levels ranging from 30 to 250 pg/mL [6,31] compared to <10 pg/mL [14] in controls, and in chronic or persistent cases, IL-6 reached > 200 pg/mL and IL-8 exceeded 10,000 pg/mL in some cohorts. Chemokines such as MCP-1, MIG/CXCL9, and IP-10/CXCL10 also showed marked early surges, sometimes in the thousands of pg/mL, followed by declines during recovery, though elevated levels persisted in subsets with chronic arthralgia. Anti-inflammatory cytokines (IL-10, TGF-β) were variably altered, with IL-10 often reduced in chronic disease, suggesting an imbalance between pro- and anti-inflammatory responses. Longitudinal data revealed that while most cytokines declined after the acute phase, IL-6, TNF-α, IL-17, and certain chemokines remained elevated months to years post-infection [27], especially in patients with persistent rheumatologic symptoms (Table 3).

Most of the patients progressed to chronic rheumatologic disease after acute infection, with prevalence rates ranging between 15 and 70% [2] based on population and following period. Chronic presentations were heterogeneous, with persistent arthralgia (40–80%), then polyarthritis (10–30%), and inflammatory arthritis resembling rheumatoid arthritis (RA) (up to 20%). Other conditions that were reported included synovitis, tenosynovitis, bursitis, fasciitis, fibromyalgia, and systemic autoimmune diseases (e.g., SLE [28], spondylarthritis). Long-term disease was reported to persist over 5 [25]–7.7 [28] years in some studies (Table 4).

## 4. Discussion

This review found that there was a rapid increase in IL-6 and IL-8 with TNF-α, IFN-γ, and interferon-inducible chemokines (MCP-1/CCL2 and the CXCR3 ligands). The patterns are closely aligned with the clinical picture of high fever, acute polyarthralgia, and rash that characterized the included case series and cohorts. Collectively, findings suggest that IL-6/IL-8 are the most commonly and significantly elevated mediators during early disease, whereas innate-interferon axes (IFN-7/CXCL9/CXCL10) and monocyte traffic (MCP-1) are also significant. These results are consistent with a model of innate activation and recruitment of neutrophils and monocytes that has downstream outcomes of the sudden, painful synovitis of acute CHIKV disease [32].

The appearance of IL-6/IL-8 in the pooled evidence is clinically and biologically plausible. IL-6 mediates hepatic acute-phase reactions and synovial inflammation [33], and IL-8 facilitates and activates neutrophils [34], both of which can easily account for the initial articular symptoms. An earlier meta-analysis of acute CHIKV cohorts found a pro-inflammatory cytokine fingerprint of interferons and interleukins such as IL-6 and IL-8 (later extended these findings to outside the context of any individual outbreak) [8]. The strong CD8/innate activation observed in acute disease in earlier immunophenotyping experiments duly complements those of chemokines (e.g., CXCL10/IP-10, CXCL9/MIG), which confirms that interferon-stimulable pathways form the core of early pathogenesis [35].

An interesting finding of this study is the variability in interferon-inducible chemokines. Many included studies have found a high CXCL10/IP-10 in acute cases; some reports indicate similar or even lower levels when compared to controls. Heterogeneity in timing of onset to sampling, assay platforms, and past co-infection are likely contributory factors. Kinetic studies indicate that CXCL10/MIG can rise at very early times and fall off as seroconversion occurs, and samples obtained even days apart will capture different parts of that curve. This observation is consistent with previous longitudinal studies demonstrating dynamic initial highs in IP-10/MIG and later modulation with increasing neutralizing responses [15].

The second key indicator in this review is the persistence (months to years) of inflammatory mediators in individuals with persistent musculoskeletal symptoms. In the chronic phase, IL-6 and IL-8 tend to be persistently elevated compared to recovered or control groups. TNF-α may be persistently low or intermittently high; Th17-related cytokines (e.g., IL-17, sometimes also IL-27) emerge in subsets with lasting disease. This trend points to a shift from an entirely innate/interferon burst to a smoldering, more adaptive prone phenotype that maintains inflammation in the synovium. Th17 pathways in animal and human disease are increasingly implicated in arthritis caused by alphaviruses. Experimental studies have demonstrated that IL-17 alone is adequate to exacerbate joint disease, which lends a mechanistic context to findings in the chronic phase [36].

Chronic outcomes synthesis, including persistent arthralgia, frank inflammatory arthritis, and RA-like phenotypes, is projected onto long-follow-up cohorts in which many patients improve between 3 and 12 months, but a clinically significant minority report disabling joint pain over many years. The prevalence of chronic symptoms is very broad but obviously non-trivial, and there are cohorts that report persistence greater than five years. These data are in line with prospective studies of La Reunion (with residual symptoms at 15 and 36 months) and emphasize that the issue of resolution is far from universal [2,3]. Heterogeneity in rates, probably due to differences in case definitions, age structure, virus lineage, and access to care, does not necessarily contradict a core chronic signal.

Mechanistically, these research results are consistent with findings of viral antigen/RNA persistence in musculoskeletal tissues as a focus of continued immune stimulation. CHIKV RNA/antigen in synovium and macrophage-rich niches has been detected by studies in both humans and nonhuman primates months after acute infection. Macrophages are suggested as reservoirs and may contribute to the presence of low-grade inflammation that is captured by the cytokine profile of chronic-phase inflammation [4,5]. This persistent-antigen model aids in resolving the reason behind some patients developing a long-tail inflammatory arthritis despite viremia waning.

Acute-phase focus on IL-6/GM-CSF/IFN-associated chemokines can also have prognostic value. Initial data related to elevated IL-6 and GM-CSF within the acute window to subsequent persistent arthralgia, suggesting that the intensity of the initial inflammatory outburst may seed chronicity, a finding that aligns with data that emphasizes these mediators as recurrently high at presentation. This review lacked formal prognostic meta-analysis, but the convergent signal across cohorts indicates that these cytokines should be assessed through prospective analysis as potential risk factors in chronic chikungunya disease [6].

It is clinically significant how chronic chikungunya arthritis and RA overlap. Some of the included studies involve symmetric polyarthritis and involvement of small joints, as well as application of disease-modifying therapies in refractory cases. The modern literature warns that chronic chikungunya arthritis may fulfill the characterization of RA, but with different serology, extra-articular spectrum, and (in many cases) course of disease. However, RA-informed management, such as early Disease-Modifying Antirheumatic Drugs (DMARDs), could be effective in selected CHIKV-induced patients with persistent inflammatory disease [10].

Recent studies stress that chronic chikungunya disease is common enough to be a public health concern, frequently like RA in its presentation, and immunologically supported by chronic synovitis mediated by cytokines [37]. Future studies should take advantage of standardized cytokine panels (including IL-6, IL-8, GM-CSF, TNF-α, IFN-γ, CXCL9/10, and IL-17) in longitudinal studies, combine them with imaging and validated disease activity parameters, and add tissue-level virologic measurements where possible. These studies would determine the validity of using the patterns of biomarkers described in this review to determine treatment and eventually reduce the chikungunya-related disability.

The strengths of this review are its broad scope, integrating information from multiple geographical locations, times of outbreak, and patient conditions, which gives a general picture of immune signatures during acute and chronic infection of CHIKV. The use of both cytokine/chemokine profiling in addition to the clinical outcomes brings a level of richness to the method, where significant links can be made between the immunological indicators and chronic disease outcome patterns. However, a number of limitations are also noted. Some studies were small (sample size), prone to bias, with heterogeneity in study designs, patient groups, timing of sample collection, and laboratory tests. Chronic outcomes were defined inconsistently, and possible confounding by co-circulating arboviruses or underlying musculoskeletal problems was not consistently dealt with.

## 5. Conclusions

The pattern of immune activation, as well as the generation of acute illness in CHIKV infection, shows consistent patterns of elevated IL-6, IL-8, TNF-alpha, IFN-γ, and interferon-inducible chemokines. These cytokines and chemokines are raised in acute illness and are consistent with the acute manifestations of fever, rash, and polyarthritis. Notably, some patients develop chronic disease, persisting for years, and where IL-6, IL-8, TNF-α, and, in some cases, IL-17 continued to be increased, which indicates a changing immune phenotype that maintains synovial inflammation. The findings presented confirm the pathogenesis of long-term chikungunya-associated arthritis through the effects of antigenic stimulation and maladaptive host responses. In addition, the interplay between chronic chikungunya arthritis and RA highlights the clinical and therapeutic importance of cytokine-mediated mechanisms.

## Figures and Tables

**Table 1:** Characteristics of included studies and population demographics.

Author (Year)	Study Design	Sample Size	Follow-Up Duration	Mean Age (SD or Range)	No. of Male/Female
Ng et al., 2009 [14]	Retrospective	10	2 weeks post-illness	22–65 years (median 35)	10 M/0 F
Hoarau et al., 2010 [4]	Cohort	15	Up to Month 18 post-infection	Mean: Recovered = 50.3 year (SD = 13.7); Chronic = 70.7 year (SD = 15.5)	Recovered: 1 M/5 F; Chronic: 1 M/8 F
Kelvin et al., 2011 [15]	Cohort	60 (50 with CHIKV + 10 healthy controls)	Acute, 6 months, 12 months	N/A	N/A
Chow et al., 2011 [6]	Case–control	30	Acute (median 4 days), early convalescent (median 10 days), late convalescent (4–6 weeks), chronic (2–3 months)	Mean 39 years (range 23–67 years; median 36.5)	26 M/4 F
Chopra et al., 2011 [16]	Cross-sectional	CHIKV (Blood: *n* = 141); Pre-CHIKV MSK (Blood: *n* = 20); Post-CHIKV MSK (Blood: *n* = 121)	1-year post-epidemic	Majority > 45 years	54 M/74 F
Chaaithanya et al., 2011 [30]	Cohort	22 patients (6 acute, 6 recovered, 10 chronic) + 6 healthy controls	10 months	Mean 34.5 years (30.5 controls, 34.5 recovered, 41.5 chronic)	N/A
Chopra A. et al., 2012 [31]	Prospective	509 acute CHIKV cases	Up to 24 months	N/A	Male–female ratio = 1:3
Lohachanakul et al., 2012 [17]	Cross-sectional	62 enrolled (35 confirmed CHIKV: 15 severe, 20 mild; 27 non-CHIKF)	30 days (blood sampling at day 1 and day 30)	Severe CHIKF: 34.9 ± 14.3; Mild CHIKF: 38.0 ± 16.5; non-CHIKF: 37.0 ± 17.5	23 M/39 F
Schilte et al., 2013 [2]	Cohort	147	36 months	Stratified by age groups (≤35 y: 30, 36–50 y: 28, 51–60: 24, 61–70: 20, >70: 45)	73 M/74 F
Venugopalan et al., 2014 [18]	Prospective	132 cases (110 symptomatic, 22 recovered); plus 49 survey controls and 80 healthy controls	6–22 months	Median: 36 years (cases) and 41 years (healthy controls)	Symptomatic: 1:1.5; Recovered: 1:3; Healthy controls: 1:1
Reddy et al., 2014 [19]	Cohort	48 patients with CHIKV infection (and 37 healthy controls)	Up to 12 weeks in 4 patients with persistent arthralgia; otherwise, acute phase (2–10 days post-onset)	Mean 40.6 years (range 21–80); persistent arthralgia group mean 52.5 years (42–57)	31 M/17 F
Raad et al., 2016 [20]	Prospective	109	9 months	Range 22–82 years; majority (56%) between 41 and 60 years	11% M/89% F
Delgado et al., 2017 [21]	Prospective	10	12 months	48 ± 15.04 years	4 M/6 F
Chang et al., 2018 [22]	Case–control	242 (121 with chronic arthritis, 121 without joint pain)	Median 20 months post-infection	With arthritis: 49 ± 17 years; Without arthritis: 48 ± 17 years	Female: 89% in both groups (108 F and 13 M per group)
Banerjee et al., 2018 [23]	Case–control	65 (25 with persisting polyarthralgia, 15 without, 25 healthy controls)	Patients assessed post 10–30 days of infection; persisting symptoms evaluated after 10 days	Median ages: Controls 47 (23–75), Without polyarthralgia 39 (20–64), With polyarthralgia 55 (35–76)	Controls 12 M/13 F, Without polyarthralgia 6 M/9 F, With polyarthralgia 10 M/15 F
Abella et al., 2019 [24]	Cross-sectional	94	Symptoms >3 months; mean 278 ± 87.8 days since onset	57 ± 14.99 years	22 M/72 F
Cavalcanti et al., 2019 [7]	Case–control	45 patients with CHIKV + 49 healthy controls	Subacute (3–12 weeks) and chronic (>12 weeks) phases	55.2 ± 13.8 years (patients); 51 ± 8.95 years (controls)	9 M/36 F (patients); 12 M/37 F (controls)
Ninla-aesong et al., 2019 [25]	Cross-sectional	123 (63 persistent arthralgia, 30 fully recovered, 30 healthy controls)	5 years	N/A	N/A
Jacob-Nascimento et al., 2021 [26]	Cohort	253 CHIKV-positive patients + (81 OAFD controls and 15 healthy controls)	2.5 years post-infection	Median 34 years (IQR 22–44) for CHIKV patients	125 M/128 F
Yu-hen Chang et al., 2024 [27]	Cohort	40	2 years	52.6 ±14.5 years	6 M/34 F
Lozano-Parra et al., 2025 [28]	Case–control	46 febrile patients (11 cases + 35 controls Piedecuesta; 14 cases + 20 controls Capitanejo)	Capitanejo: 2.2 years; Piedecuesta: 7.7 years	Median: 33.5 years (IQR 19)	Piedecuesta cases 81.2% female vs. 40% controls; Capitanejo cases 85.7% female vs. 65% controls

Abbreviations: Chikungunya Fever (CHIKF); Chikungunya Virus (CHIKV); Musculoskeletal (Female (F); Male (M); Not Available (N/A); Other Acute Febrile Diseases (OAFDs).

**Table 2:** Acute symptoms and clinical severity.

Author (Year)	Acute Symptoms	Clinical Severity of Acute Infection
Ng et al., 2009 [14]	Fever (100%), arthralgia (90%), rash (50%), conjunctivitis (40%), GI symptoms (30%), headache (30%), eye pain (20%), back pain (20%), myalgia (10%), arthritis (10%)	5 severe, 5 non-severe
Hoarau et al., 2010 [4]	Fever (38.1 ± 1 °C), rash, arthralgia	High viral load associated with severe and chronic outcomes
Kelvin et al., 2011 [15]	Fever, rash, headache, nausea, vomiting, myalgia, arthralgia/joint pain (severe joint pain = defining feature)	Defined as severe, mild, or non-symptomatic based on questionnaire at 6 and 12 months
Chow et al., 2011 [6]	Arthralgia (60%), fever (46.7%), myalgia (46.7%), rash (43.3%); joint swelling in 4 patients	Severe in 53.3% (defined by fever > 38.5 °C, pulse > 100/min, or platelet count < 100 × 10^9^/L)
Chopra et al., 2011 [16]	Fever, joint pains, body aches	N/A
Chaaithanya et al., 2011 [30]	Fever, joint pain; some had rash, myalgia, headache	Mild to severe febrile illness with arthralgia
Chopra A. et al., 2012 [31]	Fever (95.9%), chills (75.6%), polyarthralgia (93.1%), myalgia (81.3%), skin rash (33.6%), headache (18.9%), fatigue (43.2%)	Mostly self-limiting but severe joint/musculoskeletal pain; elderly affected more severely
Autoantibodies (Anti-CCP, RF)Lohachanakul et al., 2012 [17]	Fever, myalgia, arthralgia/arthritis, rash	Mild CHIKF (fever < 38.5 °C, PR < 100, platelets > 100 × 10^9^/L, recovery ≤ 30 days); severe CHIKF (fever > 38.5 °C, PR > 100, platelets < 100 × 10^9^/L, some with prolonged arthralgia > 30 days)
Schilte et al., 2013 [2]	Febrile arthralgia	Febrile arthralgia
Venugopalan et al., 2014 [18]	High fever, severe musculoskeletal pain, polyarthralgia, headaches, fatigue, rash	Typical cases: severe febrile polyarthralgia; probable cases: milder illness
Reddy et al., 2014 [19]	Fever (100%), arthralgia (83%), myalgia (63%), headache (42%), rash (29%), GI symptoms (29%), conjunctival redness (6%)	Categorized by viral load (high vs. low)
Raad et al., 2016 [20]	Fever (89%), rash (73%), headache (69%), arthralgia (76%), myalgia (72%), back pain (61%), periarticular edema (55%), nausea (31%), vomiting (19%), mucosal bleeding (5%), asthenia (82%), cutaneous manifestations (80%), meningoencephalitis (57%)	All had persistent arthralgia; 10% polyarthritis
Delgado et al., 2017 [21]	Fever, arthralgia, myalgia, rash, arthritis, nausea, vomiting, back pain, headache, purpura, mucosal bleeding	6/10 severe (DAS-28 > 5.1); 4/10 moderate/mild
Chang et al., 2018 [22]	Fever >38 °C, severe joint pain/arthritis, acute onset erythema multiforme	Severe joint pain/arthritis
Banerjee et al., 2018 [23]	Fever, joint pain	N/A
Abella et al., 2019 [24]	Fever (96.8%), rash (98.9%), myalgia (97.9%), headache (98.9%), fatigue (98.4%), abdominal pain (96.8%), nausea/emesis (97.9%), low back pain (63.8%), conjunctivitis (31.9%), adenopathy (41.5%)	11.7% hospitalized at onset
Cavalcanti et al., 2019 [7]	All patients reported persistent joint pain	N/A
Ninla-aesong et al., 2019 [25]	High fever, severe polyarthralgia, myalgia, fatigue; typical rash and headache	N/A
Jacob-Nascimento et al., 2021 [26]	Fever, rash, headache, myalgia (92%), polyarthralgia (81%), symmetric arthralgia (82%), swollen joints (41%)	Mostly mild-to-moderate febrile disease
Yu-hen Chang et al., 2024 [27]	N/A	N/A
Lozano-Parra et al., 2025 [28]	Fever, joint pain, acute febrile syndrome	N/A

Abbreviations: Chikungunya Fever (CHIKF); Confidence Interval (CI); Disease Activity Score in 28 joints (DAS-28); Gastrointestinal (GI); Interleukin (IL); Not Available (N/A); Pulse Rate (PR); Tumor Necrosis Factor-alpha (TNF-α).

**Table 3:** Timing of cytokine measurement and cytokine concentration levels.

Author (Year)	Timing of Cytokine Measurement	Patient Cytokine Levels (pg/mL)	Control Cytokine Levels (pg/mL)	Notes/Findings
Ng et al., 2009 [14]	Acute, day 2–19 (median 4.5)	IL-6: 100–150; IL-1β: 20–30; RANTES: 2000; IL-2R, IL-5, IL-7, IL-8, IL-10, IL-15, IFN-α: 50–400; IP-10 and MIG: >10,000; Eotaxin: 50–100; HGF, FGF-basic, VEGF: 200–500; EGF: <5	IL-6: <5; IL-1β: <5; RANTES: 10,000; Eotaxin: >200; EGF: 50–100	Strong pro-inflammatory cytokine elevation; suppression of RANTES and Eotaxin
Hoarau et al., 2010 [4]	Acute (day 0–5), day 15, 6 w, 3–18 m	Chronic: TNF-α: 41 vs. Recovered: 1.5; IL-8: 37 vs. 11.3; IL-6: 11.2 vs. 9.8; IFN-γ: 757.5 vs. 1037.5; IL-12: 782.6 vs. 381 (up to 1650 at M12); IL-4: 14.5 vs. 89.5; IL-13: 14.5 vs. 49.8	No control	Chronic disease linked to persistent TNF-α, IL-8, IL-12 elevation
Kelvin et al., 2011 [15]	Acute, 6 m, 12 m	IP-10: 7000 → <1000 after seroconversion; MIG: acute high → ↓1000–10,000 fold by 6–12 m; IL-10: ↓3-fold; IL-6, CXCL9, CXCL10 higher in high IgG group	No control	Strong IP-10 and MIG elevation in acute, decline over time
Chow et al., 2011 [6]	Acute (day 4), early (day 10), late (4–6 w), chronic (2–3 m)	IL-6: 128–256 vs. recovered 32–64; GM-CSF: 128 vs. 32; IL-17: 64 (only chronic); Eotaxin: 128 vs. 512; HGF: 256 vs. 512–1024	No control	Chronic arthralgia group had higher IL-6, GM-CSF, IL-17
Chopra et al., 2011 [16]	1-year post-CHIKV	IFN-γ 8.23, CXCL-10 2.67, TNF-α 40.65, IL-13 280.7	Healthy: IFN-γ 3.1, CXCL-10 3.2, TNF-α 32.3, IL-13 181.4	IFN-γ, TNF-α, IL-13 elevated compared to controls
Chaaithanya et al., 2011 [30]	Acute (5–7 days), Chronic/Recovered (10 m)	Acute: IL-6 11.8, IL-8 754.1, MCP-1 354.9; Recovered: IL-6 0.01, IL-8 1911.6; Chronic: IL-6 63.5, IL-8 12,543.6, MCP-1 2539.8	IL-6: 0.06; IL-8: 573.7; MCP-1: 190; MIP-1α: 60.4; MIP-1β: 78.5	Chronic patients showed massive IL-6 and IL-8 rise
Chopra et al., 2012 [31]	Acute, 6–24 m follow-up	IL-6 baseline 262 (7.8 times higher); remained elevated up to 24 m	Controls baseline IL-6 low	IL-6 persistence linked to chronic disease
Lohachanakul et al., 2012 [17]	Day 1 and 30	Severe: IL-6 43, MCP-1 1561, IL-8 71.8; Mild: IL-6 27.9, MCP-1 1019, IL-8 122	Non-CHIKF: IL-6 15.7, MCP-1 413.4, IL-8 148.1	Higher MCP-1 in severe CHIKF
Schilte et al., 2013 [2]	36 m post-infection	Similar to controls	Arthralgia vs non-arthralgia: IFN-γ 0.9 vs. 0.9; IL-1α 1.4 vs. 1.4; IL-1β 2.4 vs. 2.4; IL-6 undetectable vs. 5.8; IL-17 0.9 vs. 0.9; TNF-α 2.2 vs. 2.2; MCP-1 150 vs. 170; IP-10 207 vs. 214	Minimal long-term cytokine differences
Venugopalan et al., 2014 [18]	Within 4 w (acute, subacute, extended)	IFN-α 4.7; IFN-β 38.1; IFN-γ 26.8; IP-10 66.1; IL-1β 8.2; TNF-α 107.5; MCP-1 1869; IL-4 170.5; IL-6 251; IL-10 73; IL-13 576	No control	Broad pro-inflammatory and Th2 cytokine elevation
Reddy et al., 2014 [19]	Days 2–10, follow-up 1–12 w	Peak w2: IL-1β 12.4; IL-6 34.1; IL-8 1078; MIG 739; MCP-1 687.3	IL-1β 2.7; IL-6 2.6; IL-8 4.9; MIG 31.5; MCP-1 34.9	Strong acute spike, returned near baseline
Raad et al., 2016 [20]	Mean 49 days	IL-1β, IL-2, IL-6, IL-8, IL-17, TNF-α, IFN-γ ↑ in 95%	No control	Generalized cytokine elevation
Delgado et al., 2017 [21]	Diagnosis, day10, 3 m, 12 m	IL-6: 4.5 at M12; CRP 0.35; ESR 15; RF 20	No control	Persistent IL-6 at 1 year
Chang et al., 2018 [22]	Acute	TNF-α 0.65; IL-2 0.57; IL-4 0.50; IL-13 0.80 (NS)	No control	Mild elevations only
Banerjee et al., 2018 [23]	Follow-up (10–30 days)	IL-6, IFN-γ, CXCL-9 ↑; IL-10 ↓; TGF-β1 ↑	No control	Linked to persistent polyarthralgia
Abella et al., 2019 [24]	Chronic (>3 m)	IL-6 mean 4.5; IL-17 mean 1.5	No control	IL-6 detectable in about 65%
Cavalcanti et al., 2019 [7]	Subacute (3–12 w), Chronic (>12 w)	Patients: IL-27 210, IL-17A 22, IL-29 62.5, TGF-β 40.4	Controls lower values (e.g., IL-27 62.5, IL-17A 3.9)	IL-27 and IL-17A persistently high
Ninla-aesong et al., 2019 [25]	5 years post-infection	TNF-α 1.8; IL-1β 0; IL-6 6.8; IL-8 117.5; IL-12 13.1; IFN-γ 0; MCP-1 274.5	No control	Low-grade inflammation persists long term
Jacob-Nascimento et al., 2021 [26]	Acute (≤7 days), Convalescent (15–40 days)	Acute: IL-8 90, IL-6 12, MCP-1 250, RANTES 1200, IP-10 2000, IL-1β 12, TNF 20	No control	IL-6 and IL-8 higher in acute
Yu-hen Chang et al., 2024 [27]	4 y post-infection	IL-6: 1648 (2019) → 1382 (2021); IL-10: 245 → 385; IL-12p70: 116 → 214; TNF-α: 474 → 458; IFN-γ: 441 → 366; IL-17α: 343 → 290	No control	Persistent elevation of IL-6, TNF-α, IL-17α
Lozano-Parra et al., 2025 [28]	Acute (≤7 days) and subacute (≥14 days)	Acute: IL-6 7.7; IL-8 19.1; CXCL9 7230; CXCL10 6333; Subacute: IL-6 2.6; IL-8 67.4; CXCL9 4033; CXCL10 523	Controls: IL-6 4.9; IL-8 27.3; CXCL9 7926; CXCL10 24,223	Acute cases show lower CXCL10 vs. controls

Abbreviations: Chemokine (C-C motif) ligand 2 (CCL2); Chemokine (C-C motif) ligand 5 (RANTES) (CCL5); Chikungunya Virus (CHIKV); C-reactive Protein (CRP); Chemokine (C-X-C motif) ligand 8 (CXCL8/IL-8); Chemokine (C-X-C motif) ligand 9 (MIG) (CXCL9); Chemokine (C-X-C motif) ligand 10 (IP-10) (CXCL10); Day (D); Epidermal Growth Factor (EGF); Erythrocyte Sedimentation Rate (ESR); Basic Fibroblast Growth Factor (FGF-basic); Hepatocyte Growth Factor (HGF); Interferon alpha (IFN-α); Interferon beta (IFN-β); Interferon gamma (IFN-γ); Interleukin (IL); Interquartile Range (IQR); Month (M); Monocyte Chemoattractant Protein-1 (MCP-1/CCL2); Mean Fluorescence Intensity (MFI); Monokine Induced by Gamma interferon (MIG/CXCL9); Macrophage Inflammatory Protein-1 alpha (MIP-1α); Macrophage Inflammatory Protein-1 beta (MIP-1β); Picogram per milliliter (pg/mL); Rheumatoid Factor (RF); Reactive Oxygen Species (ROS); Transforming Growth Factor beta (TGF-β); Tumor Necrosis Factor-alpha (TNF-α); Week (W); Vascular Endothelial Growth Factor (VEGF).

**Table 4:** Development of chronic rheumatologic disease.

Author (Year)	Development of Chronic Rheumatologic Disease	Type of Rheumatologic Manifestations	Duration of Chronic Symptoms
Ng et al., 2009 [14]	No (follow-up limited to 2 weeks only)	2 patients had persistent arthralgia (>2 weeks); 1 had arthritis (knee effusion)	Persistent arthralgia reported >2 weeks
Hoarau et al., 2010 [4]	Yes	Chronic relapsing arthralgia, RA-like arthritis	≥12 months, some up to 18 months
Kelvin et al., 2011 [15]	Yes	Persistent joint pain, arthralgia, arthritis (mono-/oligo-/polyarthritis, tenosynovitis, fibromyalgia-like symptoms)	Up to 12 months post-infection
Chow et al., 2011 [6]	Yes	Persistent arthralgia (joint pain), no persistent swelling	2–3 months after infection onset
Chopra et al., 2011 [16]	Yes	Osteoarthritis (48%), Non-specific arthralgia (27%), Undifferentiated inflammatory arthritis (15%), Others (2%)	≥1 year
Chaaithanya et al., 2011 [30]	Yes, (only in 10/22 patients)	Chronic joint pain/arthritis resembling RA	Up to 10 months
Chopra A. et al., 2012 [31]	Yes	Predominantly non-specific arthralgias (NSA); undifferentiated inflammatory arthritis rare (0.3% at 1 year, 0.07% at 2 years)	1 year: 4.1% prevalence; 2 years: 1.6% prevalence (chronic musculoskeletal pain)
Lohachanakul et al., 2012 [17]	Yes—6/15 severe CHIKF patients had prolonged arthralgia (>30 days)	Prolonged arthralgia/arthritis; 1 case chronic arthritis	>30 days (for prolonged cases)
Schilte et al., 2013 [2]	Yes (60% had long-term arthralgia at 36 months)	Predominantly symmetrical polyarthralgia (70%)	Up to 36 months post-infection
Venugopalan et al., 2014 [18]	Yes—some cases had persistent MSK symptoms beyond 1 month	Persistent musculoskeletal pain, arthritis, polyarthralgia	Extended symptomatic phase: 15–30 days
Reddy et al., 2014 [19]	Yes, in 4 patients with persistent arthralgia	Arthralgia (persistent)	Up to 12 weeks
Raad et al., 2016 [20]	Yes (72% had persistent arthralgia and periarticular edema after 9 months)	Symmetrical polyarthritis, persistent arthralgia, periarticular edema	≥9 months follow-up, symptoms persisted
Delgado et al., 2017 [21]	Yes—2 patients developed chronic arthritis	Chronic polyarthralgia, polyarthritis, myalgias (joints: fingers, wrists, knees, ankles, toes)	>12 months (persisted at 1-year follow-up)
Chang et al., 2018 [22]	Yes (chronic arthritis/joint pain at 20 months)	Chronic arthritis (joint pain/swelling)	Up to 20 months post-infection
Banerjee et al., 2018 [23]	Yes	Chronic joint pain and inflammation (polyarthralgia)	Persisted beyond 10 days, up to months
Abella et al., 2019 [24]	Yes—all included patients had chronic rheumatologic manifestations (>3 months)	Symmetrical arthritis, synovitis (29.8%), tender/swollen joints, persistent arthralgia	Mean 278 ± 87.8 days (range: 91–365 days)
Cavalcanti et al., 2019 [7]	Yes—21 patients (46.67%) were in chronic phase (>12 weeks)	Arthritis, persistent arthralgia, swollen/painful joints	Median disease duration 12 weeks (range: 8.5–20 weeks); chronic defined as >12 weeks
Ninla-aesong et al., 2019 [25]	Yes—63/123 had persistent arthralgia (80.9% severe pain, 19.05% non-severe)	Persistent arthralgia, severe vs. non-severe joint pain, stiffness, swelling	5 years post-infection
Jacob-Nascimento et al., 2021 [26]	Yes—chronic arthralgia occurred in 42.5% (62/146)	Chronic arthralgia (polyarthralgia, symmetric arthralgia, swollen joints)	>3 months after acute infection
Yu-hen Chang et al., 2024 [27]	Yes (persistent arthritis 4–6 years post-CHIKV infection)	Persistent arthritis, mainly affecting small joints (MCP, IFP, wrists); pain, stiffness, disability	4–6 years post-infection (study follow-up adds 2 more years)
Lozano-Parra et al., 2025 [28]	Yes	Rheumatoid arthritis, spondylarthritis, SLE, post-viral arthritis, post-viral arthralgia, tenosynovitis, bursitis, fasciitis, fibromyalgia	≥3 months; up to 7.7 years follow-up

Abbreviations: Chikungunya Fever (CHKF); Chikungunya Virus (CHKV); Interphalangeal Joint of Finger (IFP); Metacarpophalangeal Joint (MCP); Musculoskeletal (MSK); Rheumatoid Arthritis (RA); Systemic Lupus Erythematosus (SLE).

## Data Availability

No new data were created or analyzed in this study. Data sharing is not applicable to this article. CHIKV Data Extraction is included in Supplementary Materials.

## Supplementary Materials

The following supporting information can be downloaded at: https://www.mdpi.com/article/10.3390/jcm14196720/s1, CHIKV Data Extraction.
